# Machine learning models can predict cancer-associated disseminated intravascular coagulation in critically ill colorectal cancer patients

**DOI:** 10.3389/fphar.2024.1478342

**Published:** 2024-11-20

**Authors:** Li Qin, Jieling Mao, Min Gao, Jingwen Xie, Zhikun Liang, Xiaoyan Li

**Affiliations:** ^1^ Department of Pharmacy, the Sixth Affiliated Hospital, Sun Yat-sen University, Guangzhou, China; ^2^ Biomedical Innovation Center, The Sixth Affiliated Hospital, Sun Yat-sen University, Guangzhou, China; ^3^ School of Pharmaceutical Science, Sun Yat-sen University, Guangzhou, China

**Keywords:** disseminated intravascular coagulation, machine learning model, intensive care unit, colorectal cancer, anticoagulation

## Abstract

**Background:**

Due to its complex pathogenesis, the assessment of cancer-associated disseminated intravascular coagulation (DIC) is challenging. We aimed to develop a machine learning (ML) model to predict overt DIC in critically ill colorectal cancer (CRC) patients using clinical features and laboratory indicators.

**Methods:**

This retrospective study enrolled consecutive CRC patients admitted to the intensive care unit from January 2018 to December 2023. Four ML algorithms were used to construct predictive models using 5-fold cross-validation. The models’ performance in predicting overt DIC and 30-day mortality was evaluated using the area under the receiver operating characteristic curve (ROC-AUC) and Cox regression analysis. The performance of three established scoring systems, ISTH DIC-2001, ISTH DIC-2018, and JAAM DIC, was also assessed for survival prediction and served as benchmarks for model comparison.

**Results:**

A total of 2,766 patients were enrolled, with 699 (25.3%) diagnosed with overt DIC according to ISTH DIC-2001, 1,023 (36.9%) according to ISTH DIC-2018, and 662 (23.9%) according to JAAM DIC. The extreme gradient boosting (XGB) model outperformed others in DIC prediction (ROC-AUC: 0.848; 95% CI: 0.818–0.878; *p* < 0.01) and mortality prediction (ROC-AUC: 0.708; 95% CI: 0.646–0.768; *p* < 0.01). The three DIC scores predicted 30-day mortality with ROC-AUCs of 0.658 for ISTH DIC-2001, 0.692 for ISTH DIC-2018, and 0.673 for JAAM DIC.

**Conclusion:**

The results indicate that ML models, particularly the XGB model, can serve as effective tools for predicting overt DIC in critically ill CRC patients. This offers a promising approach to improving clinical decision-making in this high-risk group.

## Introduction

Clinical manifestations of disseminated intravascular coagulation (DIC) vary widely, ranging from asymptomatic patients exhibiting only mild laboratory abnormalities to critically ill individuals admitted to intensive care units (ICUs) for overt DIC characterized by multiple organ damage and/or significant hemorrhage (Levi et al., 2004; Papageorgiou et al., 2018). A primary determinant for the diagnosis of overt DIC is the underlying condition that precipitates it, often linked to diseases such as sepsis or cancer (Levi and Sivapalaratnam, 2018). However, the pathogenesis of cancer-associated DIC is complex and multifactorial and remains poorly understood (Levi, 2009).

As no single laboratory test can definitively confirm or exclude DIC, international guidelines advocate using DIC scoring systems to aid in diagnosis (Levi et al., 2009; Wada et al., 2017). Based on routinely available laboratory tests, these scoring systems include a well-recognized system developed by the International Society on Thrombosis and Haemostasis (ISTH). This system evaluates DIC using four parameters: platelet count, the international normalized ratio (INR), fibrinogen levels, and D-dimer levels, with a score of ≥5 indicating DIC (Taylor et al., 2001). In 2018, adjustments were made to the threshold values for D-dimer and cut-off points to enhance the model’s sensitivity and specificity (Suzuki et al., 2018). Alternatively, the DIC score proposed by the Japanese Association for Acute Medicine (JAAM) focuses on changes in platelet count and omits fibrinogen (Iba et al., 2016).

Both the ISTH and JAAM scoring systems are used primarily to diagnose overt DIC and, therefore, cannot predict the onset of DIC in its early stages (Gando et al., 2016). It is widely recognized that patients diagnosed with overt DIC generally have a poor prognosis during their stay in the ICU (Adelborg et al., 2021). Alarmingly, a recent study revealed that patients who show late-onset DIC, who tested negative on day one but positive on day three of an ICU stay, constitute a subgroup with particularly poor prognoses in cases of septic DIC (Matsuoka et al., 2024). Early prediction of overt DIC has significant clinical value. Moreover, the application of DIC scores to cancer patients may seem counterintuitive. In this population, the utility of the DIC score may decrease due to reduced sensitivity and specificity since parameters such as D-dimer and prothrombin time are inherently elevated (Levi, 2009). This complication poses challenges in accurately assessing the risk of cancer-associated DIC. In particular, the efficacy of DIC scoring systems in patients with solid tumors, especially within the colorectal cancer (CRC) subgroup, remains underexplored in large cohort studies (Squizzato et al., 2020).

Recent technological advancements have significantly increased the availability of time-series data in ICUs, including outputs of digital sensors, laboratory results, and electronic health records. These data are typically high-dimensional and frequently contain missing values, presenting challenges and opportunities to improve patient care (Boehm et al., 2022; Jin et al., 2022). Machine learning (ML) advances have notably improved the capability to analyze such complex datasets. This study aimed to develop an ML algorithm tailored to predict DIC in critically ill patients with CRC. Additionally, this study aimed to compare the predictive capabilities of the newly developed ML algorithm for short-term mortality with established DIC scoring systems.

## Materials and methods

### Data source

This study was conducted through a retrospective analysis of a prospectively maintained CRC database at the Sixth Affiliated Hospital, Sun Yat-sen University. We analyzed data from CRC patients admitted to the ICU between January 2018 and December 2023. Exclusion criteria included patients with pre-existing hemostatic disorders, such as liver cirrhosis or failure, hematopoietic malignancies, or those diagnosed with DIC at the time of ICU admission, in line with previous validation studies (Bakhtiari et al., 2004; Gando et al., 2006). Patients with missing data necessary to calculate the JAAM and ISTH DIC scores were also excluded. The Institutional Review Board of the Sixth Affiliated Hospital of Sun Yat-sen University approved this study, granting a waiver of informed consent due to its retrospective nature (approval No. 2024ZSLYEC-210).

### Baseline data extraction

The structured query language (SQL) was used to extract various patient-related variables. The severity of the disease was evaluated using Charlson’s comorbidity index (CCI). Laboratory examinations conducted within the first 24 h after admission to the ICU included the following parameters: red blood cell counts (RBC), white blood cell counts (WBC), platelet counts (PLT), hematocrit, hemoglobin, mean corpuscular hemoglobin (MCH), mean corpuscular hemoglobin concentration (MCHC), mean corpuscular volume (MCV), eosinophils, basophils, lymphocytes, monocytes, neutrophils, creatinine, and pH levels. Blood gases measured included partial oxygen (PaO_2_) and carbon dioxide (PaCO_2_) pressures. Electrolyte assessments covered sodium, potassium, calcium, and chloride. Additional tests measured the anion gap (AG), bicarbonate, lactate, blood urea nitrogen (BUN), glucose, total bilirubin, liver enzymes such as alanine aminotransferase (ALT), alkaline phosphatase (ALP), aspartate aminotransferase (AST), and coagulation markers including prothrombin time (PT), activated partial thromboplastin time (aPTT), INR, fibrinogen, and D-dimer. Vital signs were also recorded, including mean heart rate, mean systolic and diastolic blood pressures (MSBP, MDBP), mean respiratory rate (MRR), mean temperature, mean oxygen saturation (MOS), and urine output.

### Adjudication of the diagnosis of cancer-associated DIC

PLT, INR, D-dimer, and fibrinogen levels were extracted for all included patients. DIC scores were calculated using the algorithms detailed in Supplementary Table S1. We selected the most abnormal values for patients with multiple measurements on the same day, choosing the lowest for PLT and fibrinogen and the highest for INR. Daily repeated scoring was performed after that. A patient was classified as having an overt DIC if they registered a positive DIC score on any given day. DIC was considered cancer-associated if the patient had been diagnosed with CRC within 6 months before the diagnosis of DIC, had received any cancer treatment in the previous 6 months, or had recurrent/metastatic cancer.

All medical records, laboratory results, microbiological findings, imaging results, and DIC scores were reviewed by all authors to confirm the diagnosis of cancer-associated DIC. Two authors (Z.L. and L.Q.) independently assessed whether a definitive diagnosis of DIC should be established. Disagreements were resolved by consensus. In the cases where consensus was not achievable, the opinion of a hematologist (X.L.) was sought.

### Study design

**FIGURE 1:**
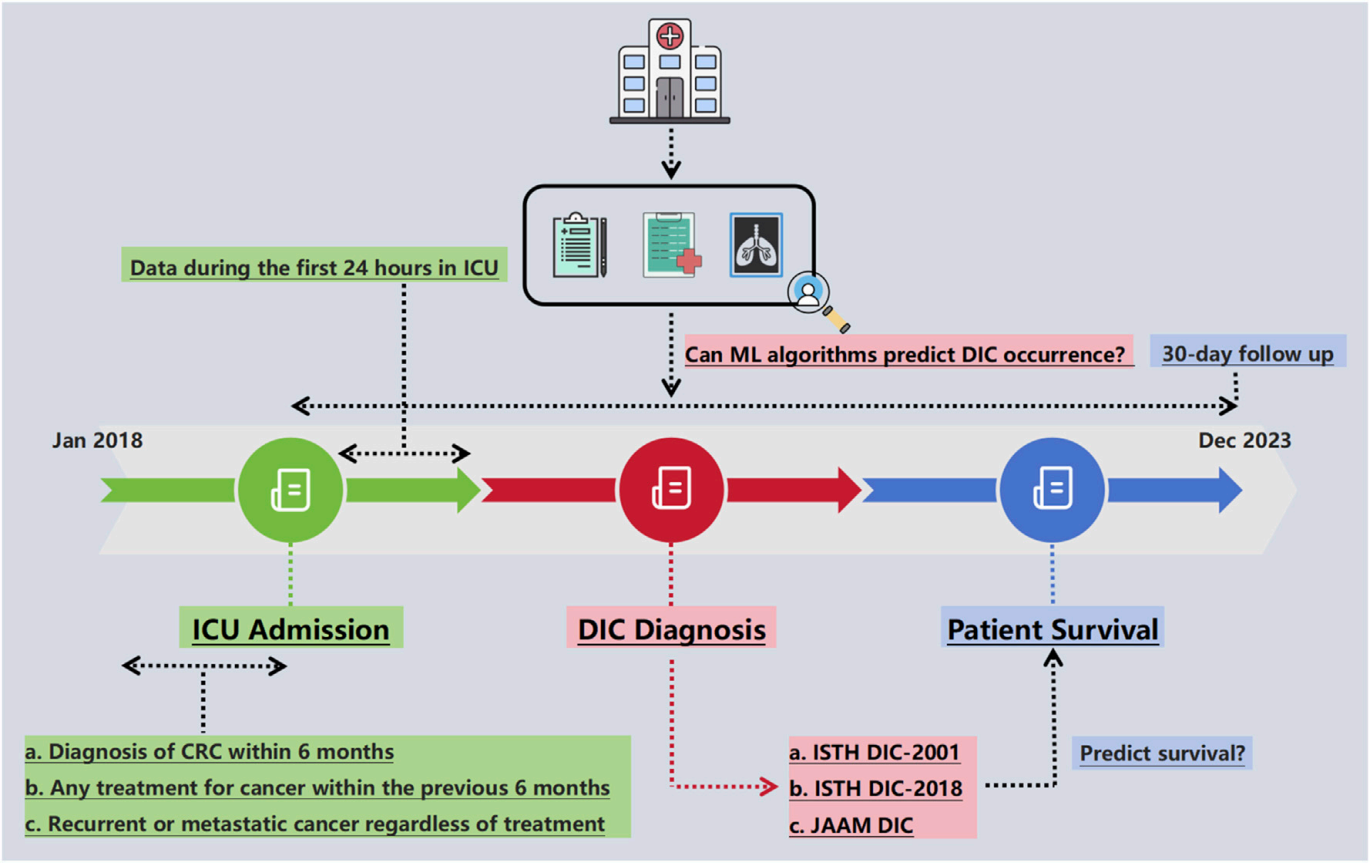
Schematic of the study design. CRC, colorectal cancer; DIC, disseminated intravascular coagulation; ICU, intensive care unit; ISTH, International Society on Thrombosis and Haemostasis; JAAM, Japanese Association for Acute Medicine; ML, machine learning.

To assess the effectiveness of DIC diagnosis, a Cox proportional hazards regression analysis was conducted to evaluate the impact of an overt DIC diagnosis, based on three different DIC scores, on mortality. This analysis adjusted for variables such as age, gender, and cancer-related factors. Following the analysis, the ISTH DIC-2018 score was selected as a criterion for outcome measurement in developing and evaluating subsequent ML models (Figure 1).

For ML model development, the patient data set was randomly divided into a training cohort (70%) and a validation cohort (30%). Continuous variables were normalized, and categorical variables were transformed into dummy variables before training. Laboratory records and vital signs were averaged with multiple daily measurements. A variety of ML models, including logistic regression (LR), random forest (RF), extreme gradient boosting (XGB), and weighted support vector machine (SVM), were utilized for DIC prediction. A 5-fold cross-validation grid search strategy was implemented for hyperparameter tuning and training in the training cohort.

The efficacy of DIC and mortality predictions was assessed across the ML models, while the performance of the three conventional scores was specifically evaluated for mortality prediction. The predictive capabilities of all scores and models were quantified using discrimination and goodness-of-fit measures. Discrimination was determined by the C-index, analogous to the ROC-AUC value for binary classification tasks. The goodness of fit was assessed using the F1 score. Additionally, the sensitivity (SEN), specificity (SPE), positive predictive value (PPV), and negative predictive value (NPV) were calculated, each with 95% confidence intervals (95% CIs). Survival probabilities for different groups, stratified by various scores and ML models, were visualized using Kaplan-Meier curves and compared using the log-rank test.

### Net reclassification improvement

Net reclassification improvement (NRI) was utilized to assess changes in the four domains of the confusion matrix when 30-day mortality was designated as the outcome. This measure was calculated by adding the improvements in sensitivity and specificity. The NRI for the XGB model was calculated and compared with three conventional DIC scores in terms of changes in sensitivity and specificity using the validation set.

### Statistical analysis

Continuous variables were presented as means with standard deviations for normally distributed data, with group comparisons performed using the Student’s t-test. Medians and interquartile ranges were reported for skewed data, and comparisons between groups were conducted using the Mann-Whitney U test. Discrete variables were summarized as frequencies and percentages, and group comparisons were made using the chi-squared or Fisher’s exact test, as appropriate. Missing data were addressed using Python’s multivariate imputation by chained equations (MICE) method.

All *p*-values are two-tailed, and a threshold of less than 0.05 was set for statistical significance. Data analysis was done using SPSS version 20.0 (IBM, Armonk, NY, United States) and the R statistical package (https://cran.r-project.org). Additionally, a SHapley Additive exPlanations (SHAP) analysis was performed to enhance model interpretability. SHAP values were calculated for each patient in the training cohort to assess the impact of individual variables on the model’s predictions. The mean absolute SHAP values across the ML models were used to globally rank variables based on their contribution to the predicted occurrence of DIC at the group level.

## Results

### Patient characteristics

The division of the data set and the study flow are shown in Supplementary Figure S1. For this analysis, 1,936 patients were selected for model development, and 830 patients were designated for external validation. Patients were categorized into DIC and non-DIC groups based on three diagnostic scores: ISTH DIC-2001, ISTH DIC-2018, and JAAM DIC. The baseline characteristics of these groups are detailed in Supplementary Table S2. Most of the patients, approximately 90%, were admitted from the emergency department or a hospital ward. Patients with stage IV cancer or those who underwent ongoing/recent chemotherapy had higher rates of DIC. In comparison, those who underwent recent surgery or underwent ongoing/recent radiotherapy had lower DIC rates. The use of bevacizumab showed negligible differences between the DIC and non-DIC groups. Follow-up durations were comparable across all groups; for instance, in the ISTH DIC-2018 group, the 75th quantile was 27 days, the 50th quantile was 16 days, and the 25th quantile was 9 days; similarly, in the non-ISTH DIC-2018 group, the durations were 19 days, 12 days, and 9 days respectively for the exact quantiles.

### Model performance comparisons

**FIGURE 2:**
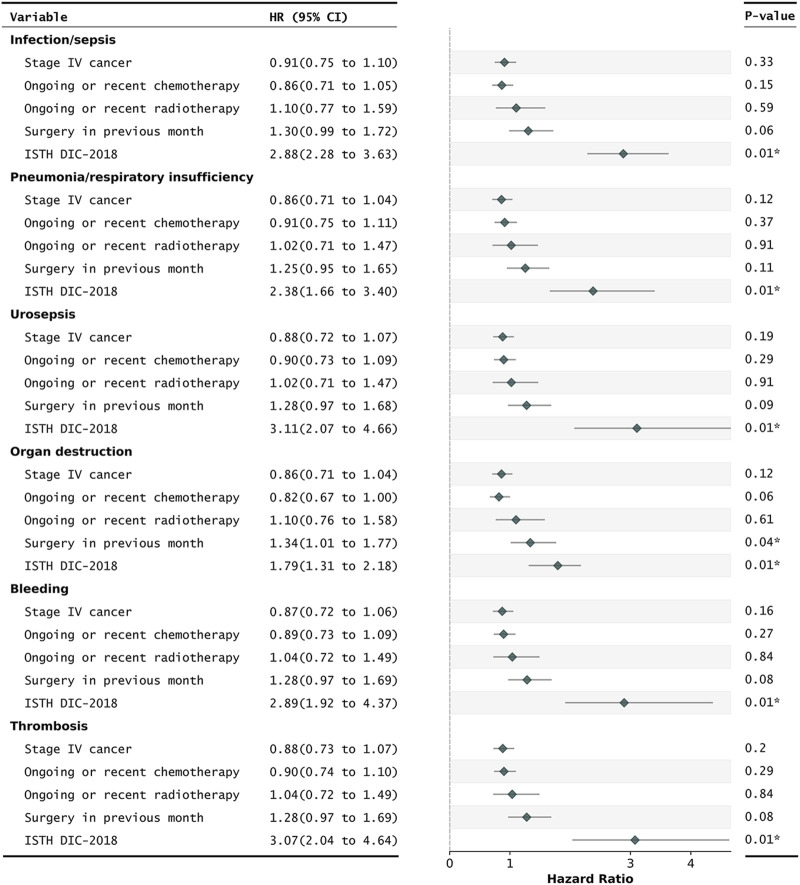
Cox regression for the prediction of 30-day mortality in critically ill CRC patients with different DIC underlying mechanisms. ISTH DIC-2018, DIC score using cut-off scores published in 2018. Daily repeated scoring was performed during ICU stays. A patient was annotated as overt DIC if they had a positive DIC score that day. All HRs were adjusted for age and gender. CI, confidence intervals; CRC, colorectal cancer; DIC, disseminated intravascular coagulation; HRs, hazard ratios; ISTH, International Society on Thrombosis and Haemostasis. *P-value of less than 0.05.

**FIGURE 3:**
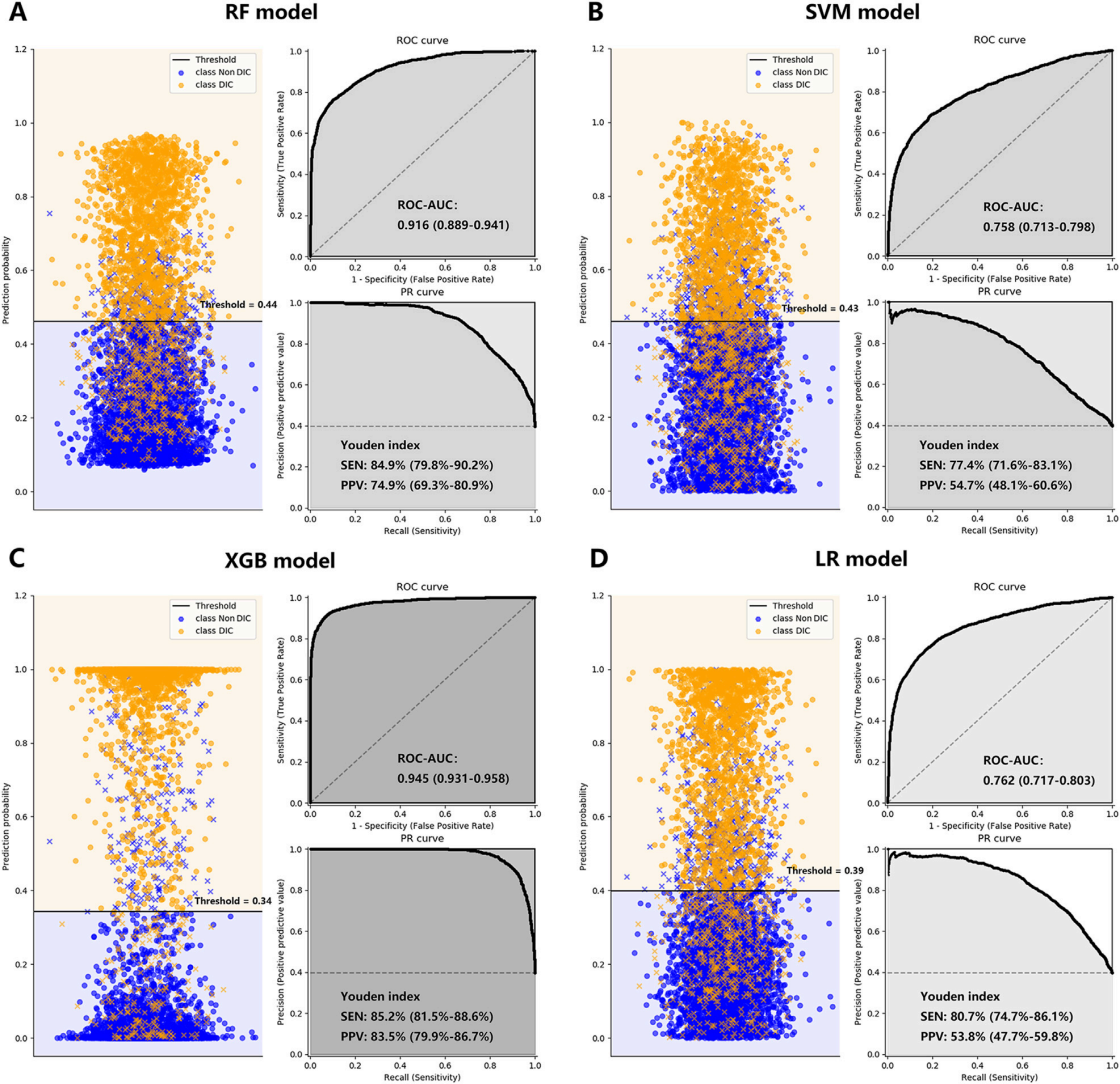
The performance of the RF Plot **(A)**, SVM Plot **(B)**, XGB Plot **(C)**, and LR Plot **(D)** models in the training cohort, with DIC diagnosed by ISTH DIC-2018 as the outcome. To evaluate the performance of the four machine learning models, we plotted the classification based on the optimal threshold and ROC and PR curves. AUCs were also calculated with 95% CIs. The optimal threshold points of the PR curves were plotted, along with their respective sensitivities and positive predictive values. CI, confidence interval; DIC, disseminated intravascular coagulation; ISTH, International Society on Thrombosis and Haemostasis; LR, logistic regression; PPV, positive predictive value; PR, precision-recall curve; RF, random forest; ROC-AUC, the area under the receiver operating characteristic curve; SEN, sensitivity; SVM, supporting vector machine; XGB, XGBoost; Youden index: = sensitivity + specificity - 1.

**FIGURE 4:**
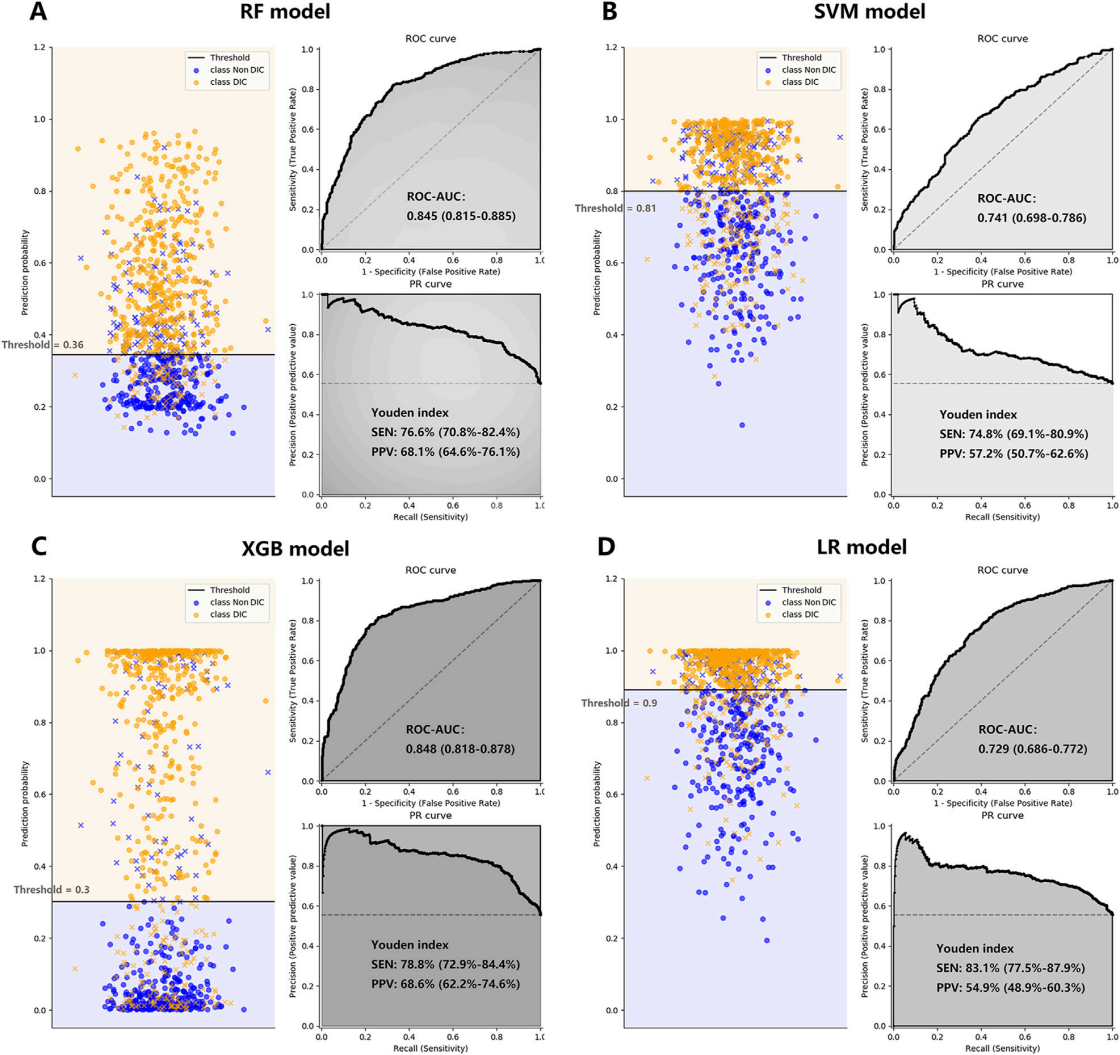
The performance of the RF Plot **(A)**, SVM Plot **(B)**, XGB Plot **(C)**, and LR Plot **(D)** models in the validation cohort, with DIC diagnosed by ISTH DIC-2018 as the outcome. To evaluate the performance of the four machine learning models, we plotted the classification based on the optimal threshold, and the ROC and PR curves. AUCs were also calculated with 95% CIs. The optimal threshold points of the PR curves were plotted, along with their respective sensitivities and positive predictive values. CI, confidence interval; DIC, disseminated intravascular coagulation; ISTH, International Society on Thrombosis and Haemostasis; LR, logistic regression; PPV, positive predictive value; PR, precision-recall curve; RF, random forest; ROC-AUC, the area under the receiver operating characteristic curve; SEN, sensitivity; SVM, supporting vector machine; XGB, XGBoost; Youden index: = sensitivity + specificity - 1.

After controlling for age, gender, and cancer-related factors, a diagnosis of DIC based on three different scores (ISTH DIC-2001, ISTH DIC-2018, and JAAM DIC) remained significantly associated with 30-day mortality in CRC patients, despite various underlying DIC mechanisms (Figure 2; Supplementary Figures S2, S3). The performance evaluations for DIC prediction using four established ML models, RF, SVM, XGB, and LR, are detailed in Table 1. Figures 3, 4 further delineate the DIC predictions of these ML models within the training and validation cohorts.

**TABLE 1:** Performance of different ML models in predicting DIC occurrence of critically ill CRC patients.

Variables (model)	AUC/C-index^a^ (95% CI)	F1 score^c^ (%)	Sensitivity (%) (95% CI)	Specificity (%) (95% CI)	Positive predictive value (%) (95% CI)	Negative predictive value (%) (95% CI)
Training cohort						
RF model^b^	0.916 (0.889–0.941)	79.6	84.9 (79.8–90.2)	83.9 (79.7–87.9)	74.9 (69.3–80.9)	90.8 (87.4–93.9)
SVM model^b^	0.758 (0.713–0.798)	64.1	77.4 (71.6–83.1)	63.9 (58.8–68.5)	54.7 (48.1–60.6)	83.4 (78.2–87.7)
XGB model^b^	0.945 (0.931–0.958)	84.3	85.2 (81.5–88.6)	88.9 (86.4–91.3)	83.5 (79.9–86.7)	90.1 (87.6–92.5)
LR model^b^	0.762 (0.717–0.803)	64.6	80.7 (74.7–86.1)	60.9 (55.7–66.2)	53.8 (47.7–59.8)	84.8 (80.2–89.5)
Validation cohort						
RF model^b^	0.845 (0.815–0.885)	72.1	76.6 (70.8–82.4)	76.0 (73.1–83.7)	68.1 (64.6–76.1)	84.0 (79.7–87.9)
SVM model^b^	0.741 (0.698–0.786)	64.8	74.8 (69.1–80.9)	63.9 (58.3–69.4)	57.2 (50.7–62.6)	79.8 (74.5–84.9)
XGB model^b^	0.848 (0.818–0.878)	73.3	78.8 (72.9–84.4)	76.8 (72.4–81.5)	68.6 (62.2–74.6)	84.9 (80.5–89.2)
LR model^b^	0.729 (0.686–0.772)	66.1	83.1 (77.5–87.9)	56.0 (50.4–61.6)	54.9 (48.9–60.3)	83.7 (78.5–88.5)

Data are ROC-AUCs, and (95% CI). AUCs, areas under the curve; CI, confidence interval; CRC, colorectal cancer; DIC, disseminated intravascular coagulation; LR, logistic regression; PPV, positive predictive value; RF, random forest; ROC, receiver-operating characteristic; SVM, supporting vector machine; XGB, XGBoost.

^a^ The value of the C-index is the same as that of AUC, in the logistic regression model.

^b^ Calculated cut-off points based on ROC, curves.

^c^ The F1 score was calculated using the formula: F1 = 2 ⋅ PPV ⋅ sensitivity/(PPV + sensitivity).

**FIGURE 5:**
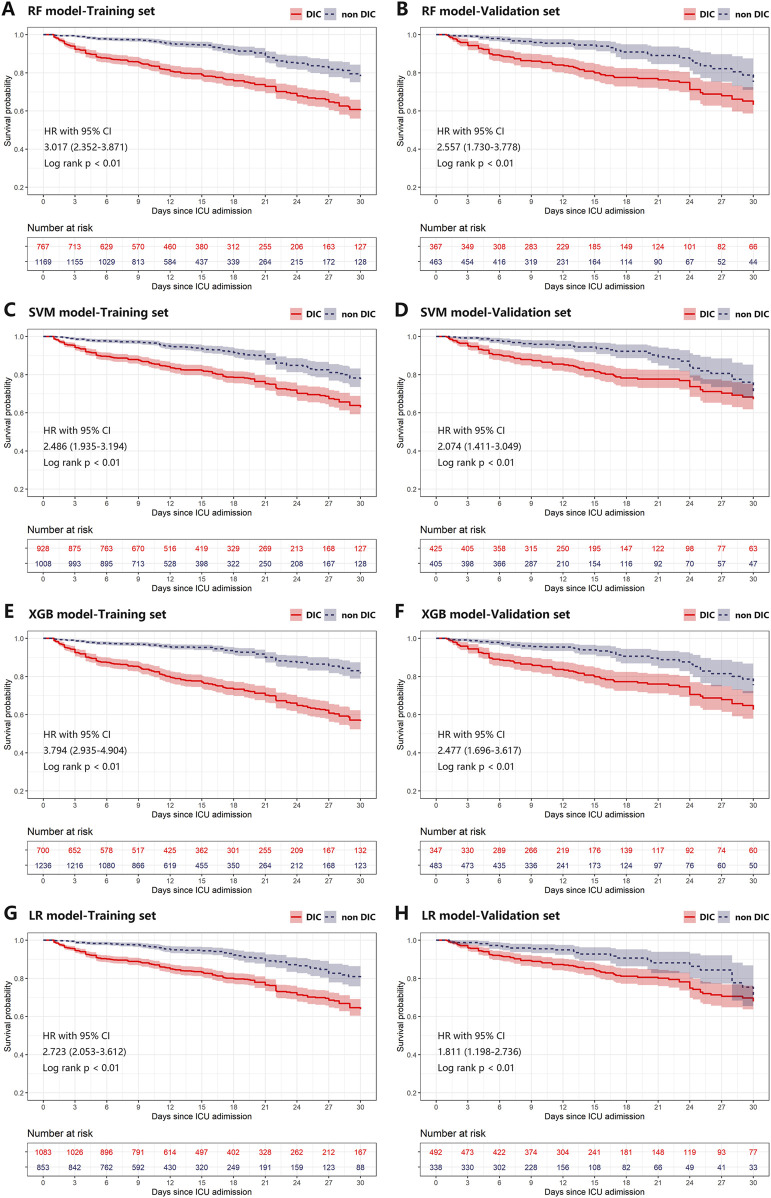
Kaplan-Meier plot of estimated 30-day mortality according to different ML models in training **(A, C, E, G)** and validation **(B, D, F, H)** cohort. Any differences in the incidence were evaluated with a log-rank test. Plot A-H was grouped by predictions of RF, SVM, XGB, and LR models on the first day of ICU stay, respectively. CI, confidence interval; DIC, disseminated intravascular coagulation; HR, hazard ratio; ICU, intensive care unit; LR, logistic regression; ML, machine learning; RF, random forest; SVM, supporting vector machine; XGB, XGBoost.

The effectiveness of all scores and ML models in predicting survival outcomes is presented in Table 2. In the training cohort, Kaplan-Meier curves revealed poorer 30-day survival among patients with positive DIC predictions, as determined by both DIC scores and ML models (Supplementary Figure S4; Figure 5). This pattern persisted in the validation cohort (Supplementary Figure S4; Figure 5). In the validation cohort, the NRI values for the 30-day mortality prediction of the XGB model compared to the DIC scores were 0.55, 0.57, and 0.54 for ISTH DIC-2001, ISTH DIC-2018, and JAAM DIC, respectively, with all corresponding *p*-values <0.01 (Table 3).

**TABLE 2:** Performance of different scores and ML models in predicting 30-day mortality of critically ill CRC patients.

Variables (model)	AUC/C-index^a^ (95% CI)	F1 score^d^ (%)	Sensitivity (%) (95% CI)	Specificity (%) (95% CI)	Positive predictive value (%) (95% CI)	Negative predictive value (%) (95% CI)
Training cohort						
ISTH DIC-2001 score^b^	0.718 (0.655–0.777)	46.8	61.8 (50.1–72.9)	81.8 (77.8–85.4)	37.7 (29.2–46.3)	92.3 (89.5–94.8)
ISTH DIC-2018 score^b^	0.712 (0.655–0.763)	42.6	72.1 (61.4–82.1)	70.4 (66.1–74.6)	30.2 (23.5–36.9)	93.4 (90.7–95.9)
JAAM DIC score^b^	0.698 (0.639–0.756)	45.2	56.3 (45.2–66.7)	83.4 (79.6–86.5)	37.7 (29.4–46.1)	91.5 (88.3–94.1)
RF model^c^	0.727 (0.661–0.791)	38.1	70.6 (60.3–79.7)	64.4 (60.1–68.7)	26.1 (20.2–32.0)	92.5 (89.6–95.3)
SVM model^c^	0.654 (0.592–0.718)	33.2	72.4 (60.8–83.2)	52.8 (48.6–57.8)	21.5 (16.4–26.9)	91.5 (87.8–94.9)
XGB model^c^	0.778 (0.724–0.825)	42.6	72.0 (62.2–81.2)	70.4 (65.9–74.9)	30.2 (24.2–36.9)	93.4 (90.6–95.8)
LR model^c^	0.734 (0.663–0.802)	33.9	77.5 (68.3–87.2)	50.2 (45.8–54.8)	21.7 (17.2–26.5)	92.6 (88.7–96.1)
Validation cohort						
ISTH DIC-2001 score^b^	0.658 (0.601–0.718)	38.4	53.2 (42.5–63.7)	78.3 (74.1–81.8)	30.1 (22.8–37.3)	90.5 (87.3–93.3)
ISTH DIC-2018 score^b^	0.692 (0.635–0.742)	39.7	71.8 (61.2–81.3)	66.6 (62.1–70.7)	27.4 (20.7–33.4)	93.1 (90.2–95.6)
JAAM DIC score^b^	0.673 (0.615–0.728)	40.3	56.5 (45.4–67.2)	78.2 (74.1–82.4)	31.3 (23.7–39.2)	91.1 (88.3–93.8)
RF model^c^	0.695 (0.628–0.751)	35.7	70.9 (60.1–80.0)	60.5 (56.0–65.1)	23.9 (18.1–28.9)	92.2 (88.9–94.9)
SVM model^c^	0.607 (0.539–0.674)	31.7	70.2 (60.7–79.9)	52.1 (47.6–56.5)	20.5 (15.7–25.2)	90.9 (87.2–94.4)
XGB model^c^	0.708 (0.646–0.768)	35.9	71.8 (61.4–81.8)	68.2 (64.5–72.3)	23.9 (18.4–29.5)	93.3 (90.4–95.8)
LR model^c^	0.670 (0.599–0.739)	30.5	75.8 (66.3–85.4)	43.6 (39.0–48.7)	19.1 (14.5–23.7)	91.1 (87.3–94.9)

Data are ROC-AUCs, and (95% CI). AUCs, areas under the curve; CI, confidence interval; CRC, colorectal cancer; DIC, disseminated intravascular coagulation; ICU, intensive care unit; ISTH, international society on thrombosis and haemostasis; JAAM, japanese association for acute medicine; LR, logistic regression; PPV, positive predictive value; RF, random forest; ROC, receiver-operating characteristic; SVM, supporting vector machine; XGB, XGBoost.

^a^ The value of the C-index is the same as that of AUC, in the logistic regression model.

^b^ Recommended cut-off points based on derivation studies.

^c^ Calculated cut-off points based on ROC, curves.

^d^ The F1 score was calculated using the formula: F1 = 2 ⋅ PPV ⋅ sensitivity/(PPV + sensitivity).

**TABLE 3:** Changes to reclassification across XGB models and existing DIC scores in 30-day mortality outcome.

From ISTH DIC-2001 to XGB model						
30-day mortality	Predicted by ISTH DIC-2001 score^a^	Predicted by XGB model^b^		Reclassified		
		Negative	Positive	Increased risk	Decreased risk	Net correctly reclassified
Mortality (n = 124)						
	<5	31	27	21.77%		14.51%
	≥5	9	57		7.26%	
Non mortality (n = 706)						
	<5	399	154	21.81%		−15.58%
	≥5	44	109		6.23%	
NRI (95% CI)						0.55 (0.40–0.75) *p* < 0.01

From ISTH DIC-2018 to XGB model						
30-day mortality	Predicted by ISTH DIC-2018 score^a^	Predicted by XGB model^b^		Reclassified		
		Negative	Positive	Increased risk	Decreased risk	Net correctly reclassified
Mortality (n = 124)						
	<4	25	10	8.06%		−4.03%
	≥4	15	74		12.09%	
Non mortality (n = 706)						
	<4	381	89	12.61%		3.83%
	≥4	62	174		8.78%	
NRI (95% CI)						0.57 (0.41–0.73) *p* < 0.01

From JAAM DIC to XGB model						
30-day mortality	Predicted by JAAM DIC score^a^	Predicted by XGB model^b^		Reclassified		
		Negative	Positive	Increased risk	Decreased risk	Net correctly reclassified
Mortality (n = 124)						
	<4	27	27	21.77%		11.29%
	≥4	13	57		10.48%	
Non mortality (n = 706)						
	<4	398	154	21.81%		−15.44%
	≥4	45	109		6.37%	
NRI (95% CI)						0.54 (0.37–0.71) *p* < 0.01

CI, confidence interval; DIC, disseminated intravascular coagulation; ICU, intensive care unit; ISTH, international society on thrombosis and haemostasis; JAAM, japanese association for acute medicine; NRI, net reclassification improvement; XGB, XGBoost.

^a^ Recmmonded cut-off points based on derivation studies.

^b^ Calculated cut-off points based on ROC, curves.

### Explainability

**FIGURE 6:**
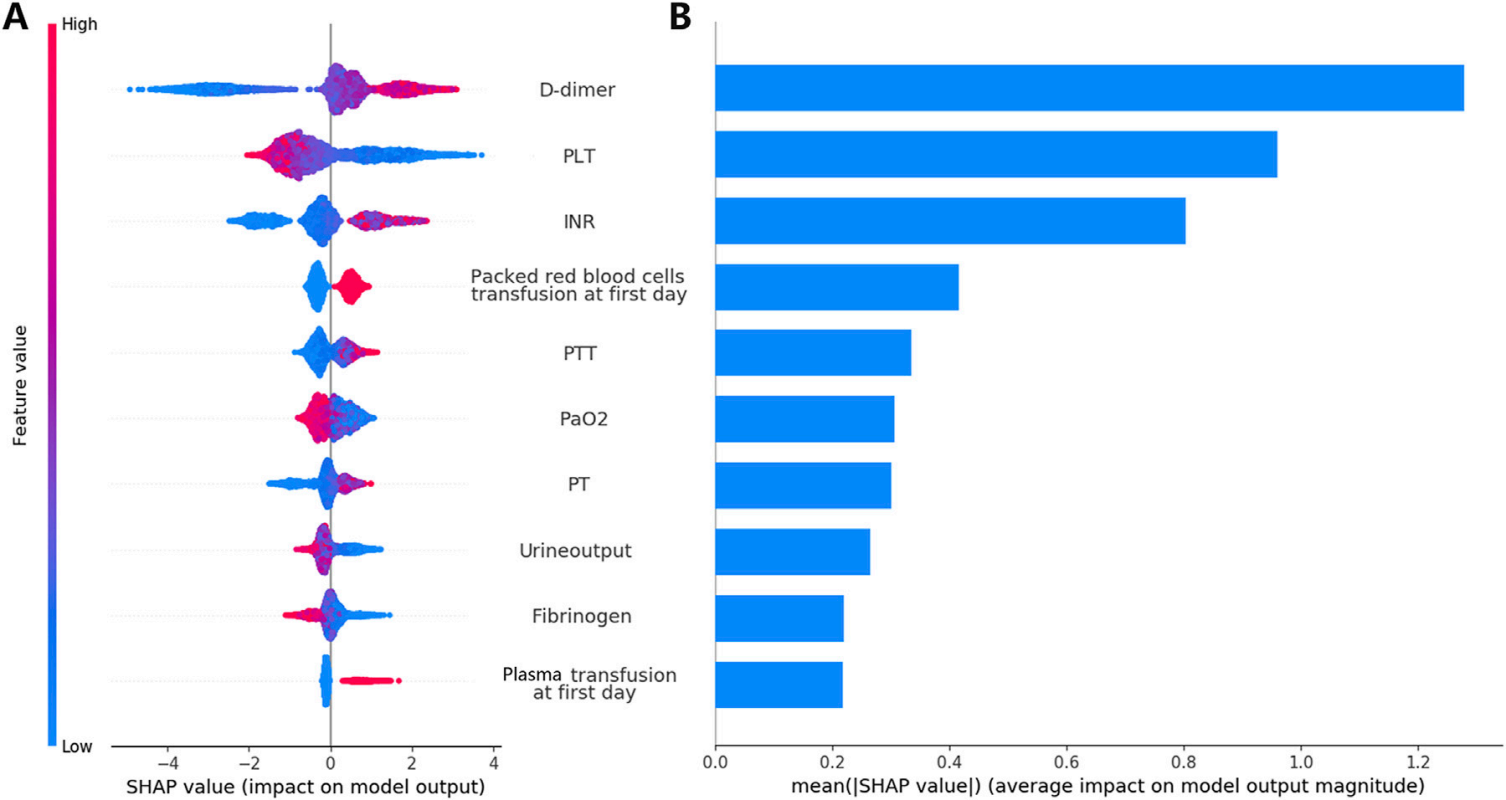
Analysis and interpretation of the XGB Model. Plot **(A)** displays the SHAP analysis results for the model training set. The variables are characterized by their mean absolute SHAP values. Distributions of the top ten ranked variables are displayed across individual patients. Each point in the figure denotes the SHAP value for a particular patient. The *y*-axis shows the ranking of each variable’s impact on the model prediction. The *x*-axis displays the SHAP value. Blue indicates lower variable values, while red indicates higher values. Plot **(B)** illustrates the average contribution of each feature to the model output as determined by the SHAP analysis. INR, international normalized ratio; PLT, platelet counts; PT, prothrombin time; PTT, activated partial thromboplastin time; SHAP, Shapley Additive Explanations; XGB, XGBoost.

Feature importance was assessed using SHAP analysis, which identified the top 10 clinical variables influencing the predictions of the XGB model (Figure 6B). Additionally, Figure 6A shows how each feature correlates with the model’s output. For instance, the SHAP values for D-dimer levels displayed an asymmetric distribution, where higher levels were strongly associated with an increased likelihood of DIC occurrence. In Supplementary Figure S5, we further detailed the impact of continuous variables on the model, including the thresholds for positive and negative SHAP values that influence the predictions.

For individual patient predictions, the final model output and the confidence in the prediction are determined by summing the SHAP contributions of each feature. Supplementary Figure S6 presents the XGB model’s predictions for three patients: one with a significant positive outcome, one with an indeterminate outcome, and one with a significant negative outcome.

## Discussion

This proof-of-concept study evaluated the efficacy of using clinical data on the first day of admission to the ICU to assess the risk of cancer-associated DIC in CRC patients. We used ML techniques to address this issue and developed four models to predict the overt DIC. Additionally, the performance of three conventional DIC scores in predicting 30-day mortality was assessed and used as benchmarks for model comparison. The XGB model was the most effective, demonstrating superior performance in predicting DIC occurrence and mortality within our validation cohort. A feature importance analysis through the XGB model highlighted the top 10 clinical variables that most significantly influence DIC prediction. These variables, ranked by impact, include D-dimer, PLT, INR, packed red blood cell transfusion, PTT, PaO_2_, PT, urine output, fibrinogen, and fresh frozen plasma transfusion. The analysis also explored how these variables specifically affect the model predictions.

### Epidemiology of overt DIC in critically ill patients with CRC

Previous research has shown that the incidence of overt DIC in ICU patients varies depending on the underlying conditions associated with DIC, including sepsis/severe infection, trauma, organ destruction, malignancy, obstetric complications, vascular abnormalities, severe hepatic failure, severe toxic or immunologic reactions, thrombosis, and bleeding (Saito et al., 2019; Larsen et al., 2021; Taylor et al., 2001; Iba et al., 2019a). While numerous studies have explored the epidemiology of overt DIC using standardized diagnostic criteria, most have focused mainly on septic patients. For instance, Ogura et al. (2014) reported that among 634 patients with severe sepsis, 46.8% were diagnosed with overt DIC using the JAAM DIC score. Similarly, in Denmark, infection/sepsis was identified as the most common cause of overt DIC, accounting for 87% of cases in a large cohort of ICU patients (Larsen et al., 2021).

Other specific conditions also present notable rates of overt DIC: Grafeneder et al. (2022) found that among patients with liver disease and low fibrinogen levels, 21.83% and 25.06% were diagnosed using ISTH DIC-2001 and ISTH DIC-2018 scores, respectively. In patients with acute type A aortic dissection undergoing artificial graft replacement, 26.6% were diagnosed using the JAAM score (Arima et al., 2022). However, a study by Grafeneder et al. (2020) that excluded septic patients and those undergoing routine surgeries or procedures in the ICU identified lower rates of DIC, with 9.6% and 12.0% of patients diagnosed using the ISTH DIC-2001 and ISTH DIC-2018 scores, respectively.

Our dataset, which included critically ill CRC patients without preexisting hemostatic disorders or liver cirrhosis, revealed moderate rates of overt DIC: 25.27% with ISTH DIC-2001, 36.98% with ISTH DIC-2018, and 23.93% with the JAAM DIC. The higher incidence of DIC diagnosed by ISTH DIC-2018 is likely due to a reduction in the model threshold from 5 to 4, enhancing sensitivity, and the allocation of up to three points for elevated D-dimer levels, which are commonly observed in cancer patients (Pabinger et al., 2013). Unfortunately, there remains a lack of published studies on the epidemiology of DIC in patients with solid tumors, highlighting a gap in the current literature.

### Significance of DIC scores and ML models

The efficacy of DIC models is typically evaluated in two domains: predicting DIC occurrence and forecasting mortality outcomes. A study by Larsen et al. (2021) focused on the predictive value (PPV) of the first-time ISTH DIC-2001 score ≥5 in internal medicine wards/ICU settings, where an expert panel established the gold standard diagnosis of DIC based on clinical and laboratory data. The overall PPV for overt DIC was 68% (95% CI: 61%–74%), which increased with higher scores, 47% (95% CI: 35%–59%) for a score of 5, escalating to 88% (95% CI: 79%–94%) for scores ≥7. In particular, PPV was higher in patients with sepsis (70%) compared to those with malignancy (40%).

Helms et al. (2020) observed a moderate sensitivity of 67% for the initial ISTH DIC-2001 score assessed within 12 h of ICU admission in patients with septic shock. Similarly, Kim et al. (2022) reported a sensitivity of 63% in a retrospective emergency department cohort. The RF and XGB models showed satisfactory DIC prediction performance in our study. We selected the XGB model for further analysis due to its high sensitivity and superior performance in the training set. In particular, even though Larsen et al. (2021) only included patients with a first-time score of ≥5, the high PPV (approximately 68% in our validation set) underlines the robustness of the model, particularly given our exclusion of patients with positive DIC scores at ICU admission. This criterion prevented us from analyzing the predictive capabilities of first-time DIC scores.

Previous research indicates that patients diagnosed with DIC, using existing scoring systems, generally have poor clinical outcomes in various diseases (Iba et al., 2017; Iba et al., 2019b). We observed high 30-day mortality rates (32%–56%) in patients with positive DIC scores on ICU admission, aligning with other studies (Saito et al., 2019; Grafeneder et al., 2020; Grafeneder et al., 2022). Conversely, patients with negative DIC scores exhibited substantially lower mortality rates (20%–26%). Our study results (Supplementary Figure S4) are consistent with these findings. Additionally, NRI analysis and ROC-AUC evaluations suggested that the XGB model slightly improved survival predictions compared to traditional DIC scores.

The ability of the XGB model to predict using only data from the first day of ICU stay, while traditional scores require daily updates, shows its potential utility. Although there is no specific treatment for cancer-associated DIC, early diagnosis can facilitate patient stratification for clinical trial enrollment, potentially identifying those who could benefit from targeted coagulopathy treatments. Furthermore, as shown in studies by Matsuoka et al. (2024) and Kim et al. (2022), early DIC prediction is crucial for managing ICU patients, particularly those with solid tumors, by identifying individuals at risk of progressing to DIC after initial negative diagnoses.

### Interpretation of the XGB model

Among the top ten significant variables identified by SHAP analysis, RBC transfusion, and plasma transfusion were particularly noteworthy. Although few studies have directly examined the impact of these transfusions on DIC risk, substantial evidence indicates that perioperative RBC transfusions are associated with an increased risk of postoperative venous thromboembolism (VTE) in various surgical procedures, demonstrating the necessity for vigilant monitoring and management of VTE risk in transfused patients (Sheth et al., 2022; Lo et al., 2023).

A recent large propensity-matched cohort study further highlighted that patients receiving combined plasma and RBC transfusions exhibit higher risks of postoperative mortality (4.52% vs. 3.32%, risk ratio: 1.36 [95% CI, 1.24–1.49]), VTE (3.92% vs. 2.70%, risk ratio: 1.36 [1.24–1.49]), pulmonary embolism (PE) (1.94% vs. 1.33%, risk ratio: 1.46 [1.26–1.68]), and DIC (0.96% vs. 0.35%, risk ratio: 2.75 [2.15–3.53]) compared to those receiving RBC transfusions alone (Choi et al., 2024). Changes in RBC properties during long-term storage, such as increased aggregability and reduced cell membrane deformability, have increased the risk of VTE after transfusions (Goel et al., 2018).

Our findings also suggest an association between lower PaO_2_ and an increased risk of DIC occurrence. The pathology of DIC involves tissue hypoxia resulting from impaired oxygen delivery and mitochondrial dysfunction, which are influenced by damage-associated molecular patterns leading to extensive microclot formation. This pathway demonstrates the need for further research to clarify the clinical implications of these alterations in increasing DIC risk.

The satisfactory performance of the XGB model in predicting DIC, as evidenced by robust ROC-AUC scores and positive predictive values, may be attributed to the model’s sensitivity to critical thresholds for D-dimer, PLT, PT, INR, and fibrinogen levels (Supplementary Figure S5). Solid tumors can induce DIC by expressing fibrinolytic proteins, like urokinase-type (u-PA) and tissue-type plasminogen activators (t-PA), leading to prolonged PT, APTT, and a hyperfibrinolytic state (Levi, 2019). This sensitivity highlights the potential utility of the model in clinical settings to identify patients at increased risk for DIC.

### Strengths and limitations

Our study contributes valuable information on DIC research, particularly its epidemiology and evaluation of conventional DIC scores in patients with solid tumors, specifically focusing on the CRC subgroup, a demographic rarely reported in the existing literature. We used advanced ML techniques to develop predictive models from diverse data collected during ICU stays. The sophisticated computational power and fitting capabilities of ML algorithms facilitated the creation of complex models capable of capturing intricate patterns and relationships. Additionally, we used the SHAP package to make the decision-making processes of the XGB model transparent, enhancing its clinical utility by helping practitioners understand how predictions are generated.

However, the scope of the study, focusing solely on CRC patients, limits its generalizability to other solid tumor populations. The relatively low PPV in survival prediction indicates further limitations of our model. Diagnosing DIC, particularly from a specific source population, often requires supplementary validation from clinical and laboratory data to mitigate the risk of misclassification. The clinical complexity in CRC patients, who may concurrently develop conditions such as abdominal sepsis and acute gastrointestinal bleeding, complicates the identification of the precise triggers of DIC. Our findings did not reveal any association between DIC risk and cancer treatments such as chemotherapy, radiotherapy, and surgery. This absence suggests the need for future studies to rigorously examine the impact of specific cancer treatments on the incidence of cancer-associated DIC. Additionally, diagnosis of DIC using the JAAM score may be underestimated, as D-dimer levels were used for calculation instead of all fibrin-related markers.

The lack of a universally accepted standard for DIC diagnosis remains a challenge in this field. In our study, diagnoses were based on medical record entries, which could lead to an underestimation of bleeding or thrombotic events, especially in patients with abbreviated follow-up periods due to mortality.

## Conclusion

We developed an ML model using the XGB algorithm to predict the occurrence of DIC in critically ill CRC patients. The XGB model demonstrated high sensitivity and positive predictive value, indicating its potential as a complementary diagnostic tool to identify patients at high risk of developing DIC. Interpretative analysis of the model provided information on the risk factors associated with DIC in this patient population.

## Data Availability

The raw data supporting the conclusions of this article will be made available by the authors, without undue reservation.
