# Supplementary material for: Machine learning models can predict cancer-associated disseminated intravascular coagulation in critically ill colorectal cancer patients
Source: Front Pharmacol. 2024 Nov 20;15:1478342. doi: 10.3389/fphar.2024.1478342 (PMC11614659; doi:10.3389/fphar.2024.1478342)
Supplement: Supplementary file 1 [file DataSheet1.pdf]

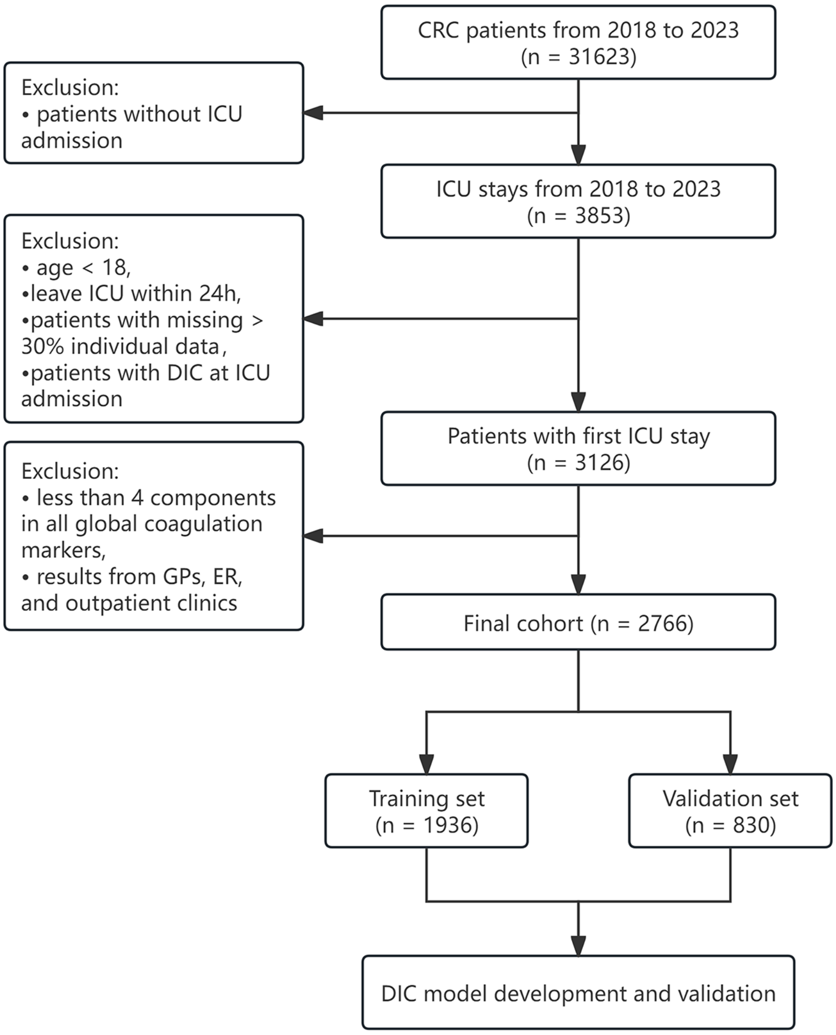

Supplementary Figure S1. Flowchart illustrating participant selection into the datasets. CRC, colorectal cancer; DIC, disseminated intravascular coagulation; ER, emergence department; ICU, intensive care unit; GP, general practice visits.

Variable	HR (95% CI)	P-value
**Infection/sepsis**		
Stage IV cancer	0.93(0.77 to 1.13)	0.48
Ongoing or recent chemotherapy	0.87(0.72 to 1.05)	0.15
Ongoing or recent radiotherapy	1.09(0.76 to 1.56)	0.63
Surgery in previous month	1.27(0.97 to 1.68)	0.09
ISTH DIC-2001	2.09(1.40 to 3.12)	0.01*
**Pneumonia/respiratory insufficiency**		
Stage IV cancer	0.87(0.72 to 1.06)	0.17
Ongoing or recent chemotherapy	0.91(0.75 to 1.10)	0.33
Ongoing or recent radiotherapy	1.03(0.72 to 1.48)	0.88
Surgery in previous month	1.26(0.96 to 1.67)	0.1
ISTH DIC-2001	1.95(1.34 to 2.84)	0.01*
**Urosepsis**		
Stage IV cancer	0.91(0.75 to 1.11)	0.35
Ongoing or recent chemotherapy	0.89(0.73 to 1.07)	0.22
Ongoing or recent radiotherapy	1.03(0.71 to 1.47)	0.89
Surgery in previous month	1.25(0.95 to 1.65)	0.11
ISTH DIC-2001	2.55(1.77 to 3.68)	0.01*
**Organ destruction**		
Stage IV cancer	0.89(0.73 to 1.08)	0.23
Ongoing or recent chemotherapy	0.81(0.66 to 0.98)	0.03*
Ongoing or recent radiotherapy	1.08(0.75 to 1.55)	0.68
Surgery in previous month	1.35(1.02 to 1.78)	0.03*
ISTH DIC-2001	2.11(1.44 to 3.08)	0.01*
**Bleeding**		
Stage IV cancer	0.90(0.74 to 1.09)	0.3
Ongoing or recent chemotherapy	0.89(0.73 to 1.07)	0.22
Ongoing or recent radiotherapy	1.05(0.73 to 1.50)	0.81
Surgery in previous month	1.25(0.95 to 1.65)	0.12
ISTH DIC-2001	2.39(1.65 to 3.45)	0.01*
**Thrombosis**		
Stage IV cancer	0.91(0.75 to 1.11)	0.36
Ongoing or recent chemotherapy	0.89(0.73 to 1.08)	0.23
Ongoing or recent radiotherapy	1.03(0.72 to 1.48)	0.86
Surgery in previous month	1.25(0.95 to 1.65)	0.11
ISTH DIC-2001	2.54(1.76 to 3.66)	0.01*

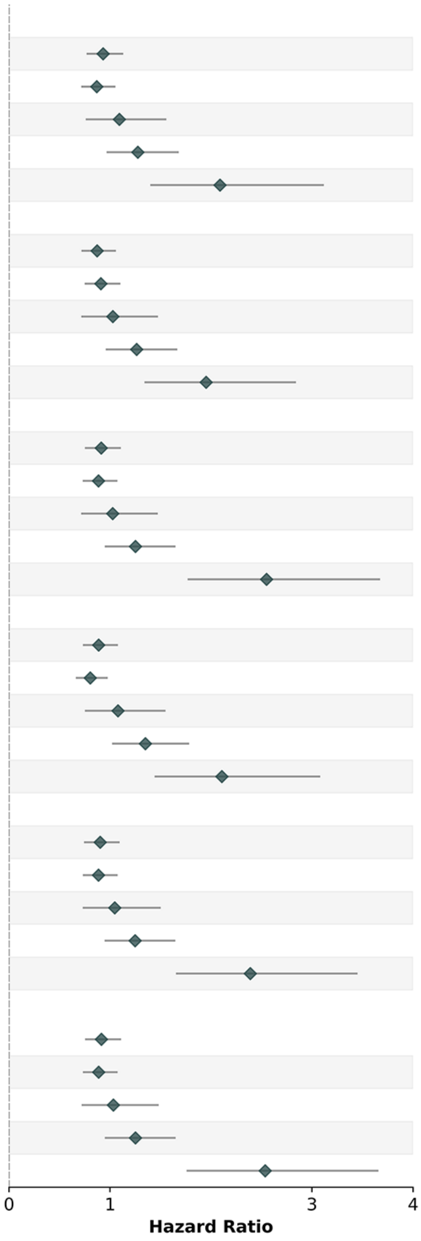

Supplementary Figure S2. Cox regression for the prediction of 30-day mortality in critically ill CRC patients with different DIC underlying mechanisms. ISTH DIC-2001, DIC score using cut-off scores published in 2001. Daily repeated scoring was performed during ICU stays. A patient was annotated as overt DIC if they had a positive DIC score that day. All HRs were adjusted for age and gender. CI, confidence intervals; CRC, colorectal cancer; DIC, disseminated intravascular coagulation; HRs, hazard ratios; ISTH, International Society on Thrombosis and Haemostasis. **P*-value of less than 0.05.

Variable	HR (95% CI)	P-value
**Infection/sepsis**		
Stage IV cancer	0.95(0.79 to 1.16)	0.64
Ongoing or recent chemotherapy	0.84(0.69 to 1.02)	0.08
Ongoing or recent radiotherapy	1.04(0.72 to 1.50)	0.83
Surgery in previous month	1.30(0.99 to 1.72)	0.06
JAAM DIC	2.79(1.88 to 4.13)	0.01*
**Pneumonia/respiratory insufficiency**		
Stage IV cancer	0.86(0.71 to 1.05)	0.14
Ongoing or recent chemotherapy	0.88(0.72 to 1.08)	0.22
Ongoing or recent radiotherapy	1.03(0.72 to 1.49)	0.85
Surgery in previous month	1.27(0.96 to 1.67)	0.09
JAAM DIC	2.49(1.71 to 3.65)	0.01*
**Urosepsis**		
Stage IV cancer	0.93(0.77 to 1.13)	0.45
Ongoing or recent chemotherapy	0.86(0.70 to 1.05)	0.13
Ongoing or recent radiotherapy	0.99(0.69 to 1.42)	0.96
Surgery in previous month	1.25(0.95 to 1.65)	0.11
JAAM DIC	3.28(2.27 to 4.73)	0.01*
**Organ destruction**		
Stage IV cancer	0.89(0.73 to 1.08)	0.25
Ongoing or recent chemotherapy	0.78(0.63 to 0.95)	0.02*
Ongoing or recent radiotherapy	1.04(0.72 to 1.49)	0.85
Surgery in previous month	1.35(1.02 to 1.78)	0.04*
JAAM DIC	2.79(1.91 to 4.07)	0.01*
**Bleeding**		
Stage IV cancer	0.92(0.76 to 1.12)	0.39
Ongoing or recent chemotherapy	0.86(0.70 to 1.05)	0.14
Ongoing or recent radiotherapy	1.01(0.71 to 1.46)	0.94
Surgery in previous month	1.23(0.93 to 1.62)	0.14
JAAM DIC	2.51(1.69 to 3.74)	0.01*
**Thrombosis**		
Stage IV cancer	0.93(0.77 to 1.13)	0.46
Ongoing or recent chemotherapy	0.86(0.71 to 1.05)	0.15
Ongoing or recent radiotherapy	1.00(0.70 to 1.44)	0.99
Surgery in previous month	1.25(0.94 to 1.64)	0.12
JAAM DIC	2.77(1.87 to 4.09)	0.01*

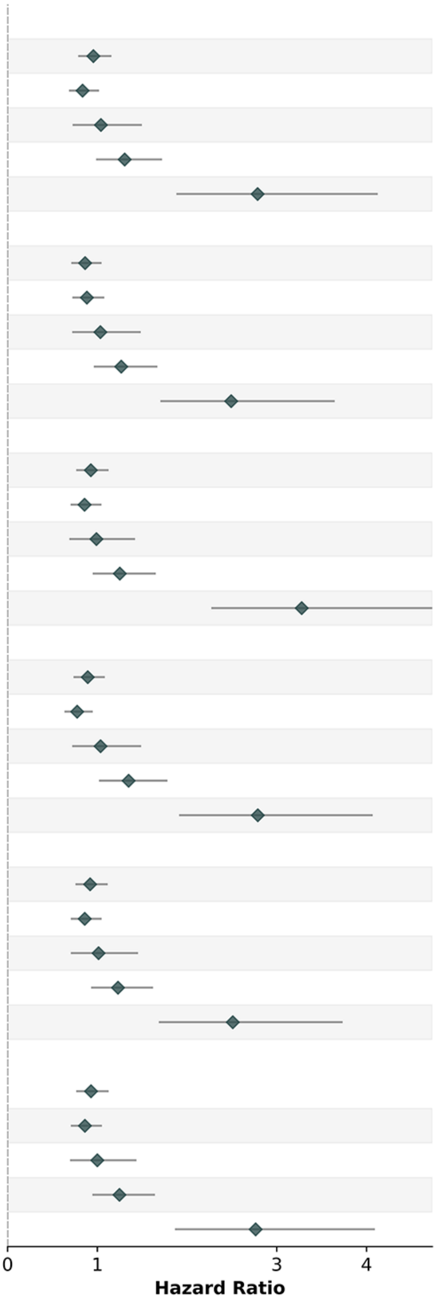

Supplementary Figure S3. Cox regression for the prediction of 30-day mortality in critically ill CRC patients with different DIC underlying mechanisms. All HRs were adjusted for age and gender. Daily repeated scoring was performed during ICU stays. A patient was annotated as overt DIC if they had a positive DIC score that day. CI, confidence intervals; CRC, colorectal cancer; DIC, disseminated intravascular coagulation; HRs, hazard ratios; JAAM, Japanese Association for Acute Medicine. **P*-value of less than 0.05.

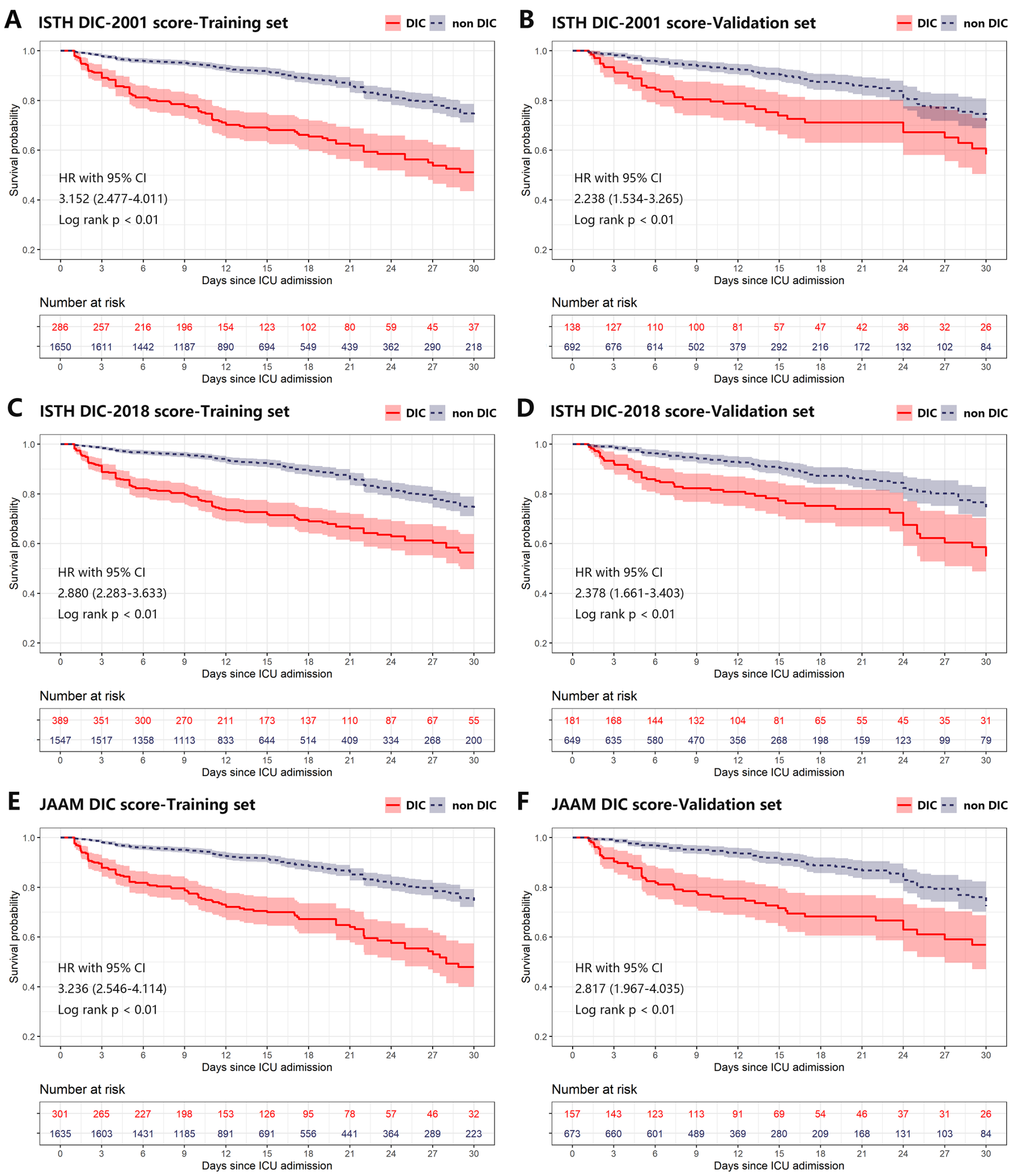

Supplementary Figure S4. Kaplan-Meier plot of estimated 30-day mortality according to different DIC scores in training and validation cohort. Any differences in the incidence were evaluated with a log-rank test. Plot A-F were grouped by ISTH DIC-2001, ISTH DIC-2018, and JAAM DIC scores calculated daily during ICU stay, respectively. A patient was annotated as overt DIC if they had a positive DIC score that day. CI, confidence interval; DIC, disseminated intravascular coagulation; HR, hazard ratio; ICU, intensive care unit; ISTH, International Society on Thrombosis and Haemostasis; JAAM, Japanese Association for Acute Medicine.

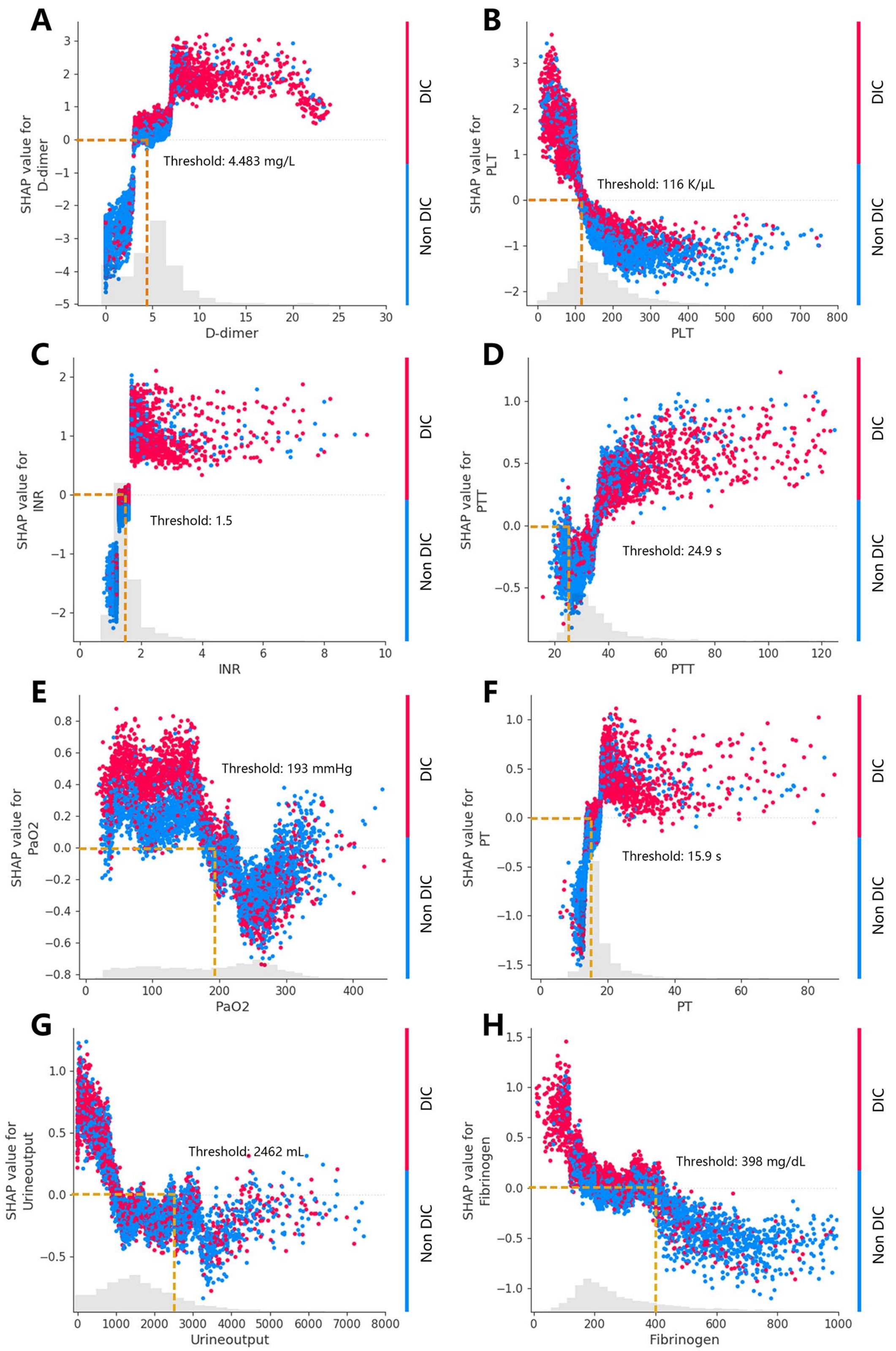

Supplementary Figure S5. SHAP values of continuous variables in the XGB model. Each plot presents SHAP values for individual patients derived from the XGB model. Each dot

represents a patient's SHAP value, with colors indicating the DIC outcome during the ICU stay. The orange line represents the threshold for distinguishing between positive and negative SHAP values. In both plots, instances with SHAP values above zero correspond to patients whose variable values push the model toward predicting DIC occurrence. In contrast, instances with SHAP values below zero correspond to patients whose variable values push the model toward predicting non-DIC occurrence. DIC, disseminated intravascular coagulation; INR, international normalized ratio; PLT, platelet counts; PT, prothrombin time; PTT, activated partial thromboplastin time; SHAP, Shapley Additive Explanations; XGB, XGBoost.

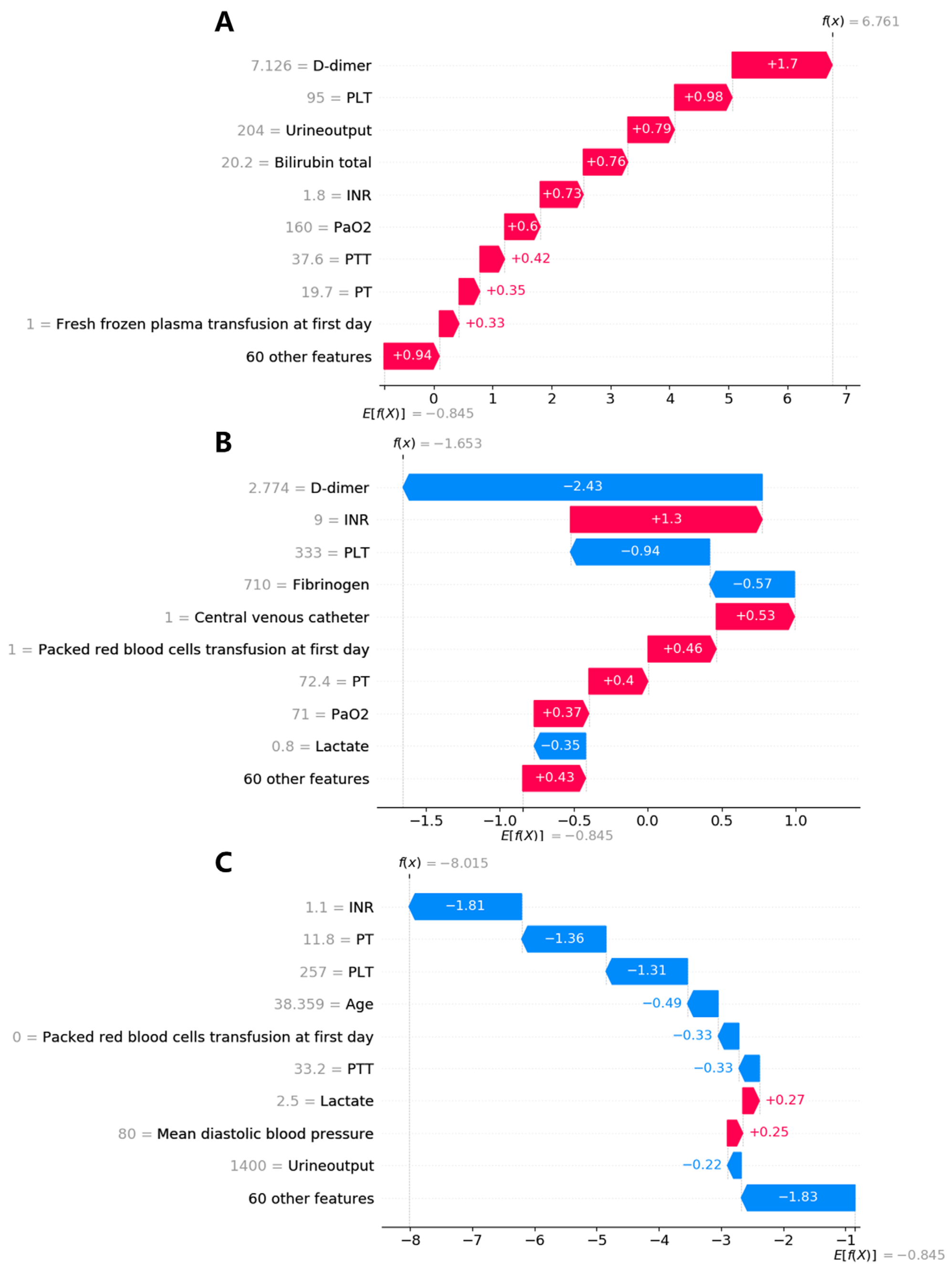

Supplementary Figure S6. Interpretation and evaluation of the XGB model. Individual predictions in the XGB model: (Plot A) a patient with a highly positive prediction, (Plot B) a patient with an indeterminate prediction, and (Plot C) a patient with a highly negative prediction. The model output for an individual patient is determined by summing the expected SHAP value (base value) and the SHAP values of all individual variables. If the model output value exceeds the expected SHAP value, it predicts DIC occurrence. Conversely, if the output

value is lower than the expected SHAP value, it predicts non-DIC occurrence. Blue indicates negative variable effects, while red indicates positive effects. The size reflects the magnitude of these effects. DIC, disseminated intravascular coagulation; INR, international normalized ratio; PLT, platelet counts; PT, prothrombin time; PTT, activated partial thromboplastin time; SHAP, Shapley Additive Explanations; XGB, XGBoost.

Supplementary Table S1. The scoring systems for DIC

	The ISTH scoring system-2001	Point	The ISTH scoring system-2018	Point	The JAAM scoring system	Point
**Platelet count (K/μL)**	< 50	2	< 50	2	< 80 or > 50% decrease within 24 hours	3
	≥ 50-100	1	≥ 50-100	1	≥ 80-120 or > 30% decrease within 24 hours	1
	> 100	0	> 100	0	≥ 120	0
**Prothrombin time (seconds)**	Prolonged prothrombin time		Prolonged prothrombin time		Value of patient/normal value	
	≥ 6	2	≥ 6	2	≥ 1.2	1
	≥ 3-6	1	≥ 3-6	1	< 1.2	0
	< 3	0	< 3	0		
**Fibrin-related**	≥ 2	3	≥ 7	3	≥ 15	3

**marker (D-dimer) (mg/L)**	≥ 1-2	2	≥ 3-7	2	≥ 5-15	1
	< 1	0	< 3	0	< 5	0
**SIRS score**					≥ 3	1
					0-2	0
**Fibrinogen level (mg/dL)**	< 100	1	< 100	1		
	≥ 100	0	≥ 100	0		
**DIC**	≥ 5 points		≥ 4 points		≥ 4 points	

DIC, disseminated intravascular coagulation; ISTH, International Society on Thrombosis and Haemostasis; JAAM, Japanese association for acute medicine; SIRS, systemic inflammatory response syndrome.

Supplementary Table S2. Patient characteristics at baseline

Patient characteristics	All patients, n = 2766	ISTH DIC-2018 patients, n = 1023	Non ISTH DIC-2018 patients, n = 1743	ISTH DIC-2001 patients, n = 699	Non ISTH DIC-2001 patients, n = 2067	JAAM DIC patients, n = 662	Non JAAM DIC patients, n = 2104
**Patient-related Factors**							
Age, mean (± SD)	65 (54 to 74)	65 (53 to 74)	65 (55 to 74)	65 (53 to 74)	65 (55 to 74)	64 (52 to 74)	66 (55 to 74)
Females, n (%)	312 (34.4)	175 (34.7)	137 (33.9)	163 (33.6)	149 (35.2)	120 (34.7)	192 (34.2)
BMI, kg/m^2, mean (± SD)	21.54 (19.03 to 24.43)	21.22 (18.83 to 24.19)	22.08 (19.16 to 24.85)	22.81 (18.73 to 23.97)	22.15 (19.49 to 24.84)	20.82 (18.81 to 24.09)	21.87 (19.14 to 24.77)
ICU admission							
Emergency department, n (%)	1294 (46.8)	448 (43.8)	846 (48.5)	327 (46.8)	967 (46.8)	323 (48.8)	971 (46.2)
Hospital ward, n (%)	1195 (43.2)	487 (47.6)	708 (40.6)	313 (44.9)	882 (42.7)	279 (42.2)	916 (43.5)
Other hospital, n (%)	277 (10.0)	88 (8.6)	189 (10.8)	59 (8.3)	218 (10.5)	60 (9.0)	217 (10.3)
CCI, median (IQR)	5 (3 to 7)	5 (4 to 7)	4 (3 to 6)	5 (4 to 7)	4 (3 to 6)	5 (4 to 7)	4 (3 to 6)

**CRC-related factors**							
Stage III, n (%)	1516 (54.8)	504 (49.3)	1012 (58.1)	342 (48.9)	1174 (56.8)	334 (50.5)	1182 (56.2)
Stage IV, n (%)	1250 (45.2)	519 (50.7)	731 (41.9)	357 (51.1)	893 (43.2)	328 (49.5)	922 (43.8)
Ongoing or recent chemotherapy, n (%)	1413 (51.1)	481 (47.0)	932 (53.5)	337 (48.2)	1076 (52.1)	316 (47.7)	1097 (52.1)
Ongoing or recent radiotherapy, n (%)	238 (8.6)	72 (7.0)	166 (9.5)	53 (7.6)	185 (9.0)	52 (7.9)	186 (8.8)
Surgery in previous month, n (%)	365 (13.2)	109 (10.7)	256 (14.7)	78 (11.2)	287 (13.9)	73 (11.0)	292 (13.9)
Bevacizumab use, n (%)	318 (11.5)	113 (11.0)	205 (11.8)	78 (11.2)	240 (11.6)	73 (11.0)	245 (11.6)
**DIC etiology**							
Infection/sepsis, n (%)	593 (21.4)	427 (41.7)	166 (9.5)	329 (47.1)	264 (12.8)	308 (46.5)	285 (13.5)
Pneumonia, n (%)	996 (36.0)	619 (60.5)	377 (21.6)	414 (59.2)	582 (28.2)	382 (57.7)	614 (29.2)
Urosepsis, n (%)	143 (5.2)	68 (6.6)	75 (4.3)	35 (5.0)	108 (5.2)	42 (6.3)	101 (4.8)
Organ destruction, n (%)	146 (5.3)	124 (12.1)	22 (1.3)	111 (15.9)	35 (1.7)	101 (15.3)	45 (2.1)
Vascular abnormalities, n (%)	94 (3.4)	50 (4.9)	44 (2.5)	28 (4.0)	66 (3.2)	26 (3.9)	68 (3.2)

Bleeding, n (%)	532 (19.2)	299 (29.2)	233 (13.4)	221 (31.6)	311 (15.0)	214 (32.3)	318 (15.1)
Thrombosis, n (%)	411 (14.9)	229 (22.4)	182 (10.4)	135 (19.3)	276 (13.4)	139 (21.0)	272 (12.9)
**Laboratory results**							
RBC counts, m/μL, mean (± SD)	2.9 (2.3 to 3.5)	2.7 (2.2 to 3.3)	3.1 (2.6 to 3.8)	2.6 (2.2 to 3.2)	3.1 (2.6 to 3.8)	2.6 (2.1 to 3.2)	3.1 (2.5 to 3.7)
WBC counts, K/μL, mean (± SD)	10.5 (7.2 to 15.7)	10.1 (6.5 to 15.5)	11.1 (7.8 to 16.3)	10.1 (6.3 to 15.5)	11.0 (7.9 to 16.1)	9.7 (6.2 to 14.9)	10.9 (7.7 to 16.3)
Platelet counts, K/μL, mean (± SD)	166 (102 to 236)	132 (70 to 208)	199 (146 to 268)	126 (67 to 204)	200 (149 to 263)	96 (50 to 159)	200 (149 to 264)
Hematocrit, %, mean (± SD)	28.4 (24.6 to 32.8)	27.6 (23.8 to 32.5)	28.8 (25.3 to 32.9)	27.6 (23.7 to 32.4)	28.8 (25.3 to 32.9)	27.6 (23.7 to 32.5)	28.8 (25.2 to 32.9)
Hemoglobin, g/dL, mean (± SD)	9.4 (8.1 to 10.9)	9.1 (7.8 to 10.7)	9.6 (8.3 to 11.0)	9.1 (7.8 to 10.7)	9.6 (8.3 to 11.0)	9.1 (7.8 to 10.7)	9.5 (8.3 to 10.9)
MCH, pg, mean (± SD)	29.5 (27.7 to	29.6 (28.1 to	29.3 (27.1 to	29.5 (28.0 to	29.3 (27.0 to	29.6 (28.2 to	29.3 (27.4 to 30.8)

	30.8)	30.9)	30.7)	30.8)	30.8)	30.9)	
MCHC, %, mean (± SD)	33.1 (32.0 to 34.1)	33.0 (31.8 to 34.0)	33.2 (32.1 to 34.2)	32.9 (31.8 to 34.0)	33.2 (32.2 to 34.2)	33.1 (32.0 to 34.1)	33.1 (32.0 to 34.1)
MCV, fL, mean (± SD)	88 (84 to 92)	89 (85 to 93)	88 (83 to 92)	88 (85 to 93)	88 (83 to 92)	89 (85 to 94)	88 (83 to 92)
Eosinophils, K/μL, mean (± SD)	0.02 (0.00 to 0.09)	0.02 (0.00 to 0.08)	0.02 (0.00 to 0.09)	0.02 (0.00 to 0.11)	0.02 (0.00 to 0.08)	0.02 (0.00 to 0.07)	0.02 (0.00 to 0.09)
Basophils, K/μL, mean (± SD)	0.01 (0.00 to 0.02)	0.01 (0.00 to 0.02)	0.01 (0.00 to 0.02)	0.01 (0.00 to 0.02)	0.01 (0.00 to 0.02)	0.01 (0.00 to 0.02)	0.01 (0.00 to 0.02)
Lymphocytes, K/μL, mean (± SD)	0.66 (0.41 to 1.01)	0.58 (0.35 to 1.03)	0.72 (0.48 to 0.98)	0.58 (0.34 to 0.99)	0.72 (0.48 to 1.01)	0.54 (0.32 to 1.02)	0.71 (0.46 to 1.01)

Monocytes, K/µL, mean (± SD)	0.47 (0.27 to 0.75)	0.44 (0.21 to 0.74)	0.50 (0.34 to 0.75)	0.44 (0.21 to 0.73)	0.49 (0.34 to 0.75)	0.41 (0.16 to 0.71)	0.51 (0.32 to 0.76)
Neutrophils, K/µL, mean (± SD)	8.59 (5.75 to 12.79)	8.14 (4.98 to 12.59)	8.97 (6.46 to 12.91)	8.14 (4.96 to 12.6)	8.98 (6.47 to 12.87)	7.67 (4.69 to 11.66)	8.99 (6.41 to 13.03)
Creatinine, mg/dL, mean (± SD)	0.9 (0.6 to 1.4)	0.9 (0.6 to 1.6)	0.8 (0.6 to 1.1)	0.9 (0.6 to 1.7)	0.8 (0.6 to 1.1)	1.1 (0.7 to 1.8)	0.8 (0.6 to 1.1)
pH, mean (± SD)	7.40 (7.35 to 7.45)	7.40 (7.35 to 7.46)	7.40 (7.36 to 7.43)	7.40 (7.35 to 7.45)	7.40 (7.36 to 7.44)	7.40 (7.33 to 7.46)	7.40 (7.36 to 7.44)
PaO_2, mmHg, mean (± SD)	147 (103 to 178)	145 (97 to 176)	148 (109 to 180)	143 (97 to 176)	148 (108 to 180)	144 (95 to 178)	147 (108 to 179)
$PaCO_2$, mmHg, mean (± SD)	36 (31 to 41)	35 (30 to 41)	37 (32 to 41)	35 (30 to 41)	37 (32 to 41)	35 (30 to 41)	37 (32 to 40)
Sodium, mEq/L, mean (± SD)	138	139	137	139	137	139	137

	(135 to 142)	(135 to 143)	(134 to 140)	(135 to 144)	(135 to 140)	(135 to 145)	(135 to 140)
Potassium, mEq/L, mean (± SD)	3.7 (3.4 to 4.1)	3.7 (3.4 to 4.1)	3.7 (3.4 to 4.1)	3.7 (3.4 to 4.1)	3.7 (3.4 to 4.1)	3.7 (3.4 to 4.1)	3.7 (3.4 to 4.1)
Calcium, mg/dL, mean (± SD)	11.5 (11.0 to 12.1)	11.4 (10.8 to 12.0)	11.6 (11.1 to 12.1)	11.5 (11.0 to 12.1)	11.6 (11.1 to 12.1)	11.4 (10.7 to 12.0)	11.6 (11.1 to 12.1)
Chloride, mEq/L, mean (± SD)	108 (105 to 112)	109 (104 to 113)	108 (105 to 110)	109 (104 to 114)	108 (105 to 110)	108 (104 to 114)	108 (105 to 111)
Aniongap, mEq/L, mean (± SD)	11 (8 to 13)	11 (9 to 15)	10 (8 to 12)	11 (9 to 15)	10 (8 to 12)	11 (9 to 16)	10 (8 to 12)
Bicarbonate, mEq/L, mean (± SD)	22 (20 to 25)	22 (19 to 25)	23 (20 to 25)	22 (19 to 25)	23 (20 to 25)	22 (18 to 25)	23 (20 to 25)
Lactate, mmol/L, mean (± SD)	1.4 (1.0 to 2.2)	1.6 (1.0 to 2.7)	1.2 (0.9 to 1.7)	1.6 (1.1 to 2.8)	1.2 (0.9 to 1.7)	1.8 (1.1 to 3.2)	1.2 (0.9 to 1.8)
BUN, mg/dL, mean (± SD)	21 (17 to 38)	25 (15 to 45)	19 (15 to 28)	24 (15 to 45)	16 (11 to 24)	23 (13 to 46)	19 (15 to 27)

Glucose, mg/dL, mean (± SD)	82 (65 to 103)	82 (64 to 103)	83 (68 to 104)	82 (64 to 106)	83 (67 to 102)	79 (64 to 105)	83 (68 to 103)
Bilirubin total, mg/dL, mean (± SD)	1.5 (1.0 to 2.5)	1.7 (1.1 to 2.9)	1.3 (0.9 to 2.0)	1.7 (1.1 to 2.9)	1.3 (0.9 to 2.0)	1.9 (1.2 to 3.3)	1.3 (0.9 to 2.1)
ALT, IU/L, mean (± SD)	16 (10 to 33)	17 (9 to 41)	14 (10 to 26)	17 (10 to 41)	15 (10 to 27)	18 (10 to 49)	15 (10 to 27)
ALP, IU/L, mean (± SD)	87 (48 to 113)	90 (53 to 122)	83 (44 to 110)	83 (46 to 111)	87 (44 to 118)	88 (49 to 122)	79 (45 to 109)
AST, IU/L, mean (± SD)	24 (17 to 54)	32 (18 to 80)	21 (15 to 34)	32 (18 to 82)	21 (15 to 35)	38 (19 to 98)	22 (16 to 37)
PT, sec, mean (± SD)	14.1 (12.5 to 16.1)	15.1 (13.2 to 17.3)	13.1 (12.1 to 14.3)	15.4 (13.7 to 17.9)	13.1 (12.1 to 14.1)	15.1 (13.2 to 17.6)	13.5 (12.3 to 15.2)
PTT, sec, mean (± SD)	32.1 (28.9 to 37.1)	33.7 (30.2 to 39.4)	30.8 (27.8 to 34.3)	34.3 (30.6 to 40.2)	30.5 (27.6 to 33.8)	34.3 (30.7 to 40.3)	31.2 (28.2 to 35.1)

INR, mean (± SD)	1.2 (1.1 to 1.4)	1.3 (1.1 to 1.5)	1.2 (1.1 to 1.2)	1.3 (1.2 to 1.5)	1.1 (1.0 to 1.2)	1.3 (1.2 to 1.5)	1.2 (1.1 to 1.3)
Fibrinogen, mg/dL, mean (± SD)	330 (250 to 410)	310 (240 to 420)	330 (280 to 408)	310 (230 to 410)	340 (280 to 420)	300 (218 to 403)	340 (280 to 420)
Fibrin D-dimer, mg/L, mean (± SD)	4.535 (1.881 to 7.595)	6.385 (3.595 to 9.473)	2.365 (1.443 to 5.295)	5.780 (2.311 to 9.224)	3.471 (1.630 to 6.191)	6.981 (3.768 to 9.393)	3.494 (1.678 to 5.943)
**Vital signs**							
Mean heart rate, min^{-1}, mean (± SD)	98 (81 to 116)	102 (86 to 121)	91 (77 to 110)	104 (88 to 122)	90 (77 to 108)	105 (90 to 123)	94 (78 to 112)
MSBP, mmHg, mean (± SD)	109 (102 to 118)	108 (101 to 117)	110 (103 to 119)	109 (101 to 117)	111 (105 to 120)	108 (100 to 116)	110 (104 to 120)
MDBP, mmHg, mean (± SD)	56 (52 to 64)	55 (50 to 63)	56 (52 to 63)	54 (50 to 62)	57 (52 to 64)	55 (50 to 62)	56 (52 to 63)
MRR, min^{-1}, mean (± SD)	20 (16 to 25)	20 (16 to 27)	18 (15 to 23)	20 (17 to 28)	18 (15 to 23)	20 (17 to 27)	19 (15 to 23)

Mean temperature, ºC, mean (± SD)	36.8 (36.5 to 37.1)	36.8 (36.5 to 37.0)	36.8 (36.5 to 37.0)	36.8 (36.5 to 37.0)	36.8 (36.5 to 37.0)	36.8 (36.5 to 37.0)	36.8 (36.5 to 37.0)
MOS, %, mean (± SD)	99.41 (98.81 to 99.81)	99.40 (98.71 to 99.81)	99.41 (98.90 to 99.91)	99.40 (98.71 to 99.81)	99.41 (98.90 to 99.91)	99.41 (98.60 to 99.73)	99.41 (98.90 to 99.91)
Urine output, mL, mean (± SD)	1547 (866 to 2299)	1400 (741 to 2134)	1748 (1104 to 2500)	1375 (740 to 2120)	1750 (1100 to 2450)	1350 (673 to 2095)	1680 (1050 to 2431)
**Treatments**							
Mechanical ventilation, n (%)	1922 (69.5)	724 (70.8)	1198 (68.7)	491 (70.3)	1431 (69.2)	474 (71.6)	1448 (68.8)
Vasopressor, n (%)	1828 (66.1)	716 (70.0)	1112 (63.8)	489 (70.0)	1339 (64.8)	461 (69.6)	1367 (65.0)
Central venous catheter, n (%)	572 (20.7)	275 (26.9)	297 (17.0)	192 (27.5)	380 (18.4)	182 (27.5)	390 (18.5)
Sedative, n (%)	133 (4.8)	85 (8.3)	48 (2.8)	58 (8.3)	75 (3.6)	58 (8.8)	75 (3.6)
Transfusion of blood product							
Platelet, n (%)	228 (8.2)	133 (13.0)	95 (5.5)	109 (15.6)	119 (5.8)	109 (16.5)	119 (5.7)

Fresh frozen plasma, n (%)	867 (31.3)	390 (38.1)	477 (27.4)	305 (43.6)	562 (27.2)	269 (40.6)	598 (28.4)
Packed red blood cells, n (%)	850 (30.7)	341 (33.3)	509 (29.2)	242 (34.6)	608 (29.4)	234 (35.3)	616 (29.3)

ALT, alanine aminotransferase; ALP, alkaline phosphatase; AST, aspartate aminotransferase; BMI, body mass index; BUN, Blood Urea Nitrogen; CCI, Charlson comorbidity index; CRC, colorectal cancer; DIC, dessiminated intiavascular coagulation; ICU, intensive care unit; INR, international normalized ratio; IQR, interquartile ranges; ISTH, International Society on Thrombosis and Haemostasis; JAAM, Japanese association for acute medicine; LMWH, low molecular weight heparin; MCH, mean corpuscular hemoglobin; MCHC, mean corpuscular hemoglobin concentration; MCV, mean corpuscular volume; MDBP, mean diastolic blood pressure; MOS, mean oxygen saturation; MRR, mean respiratory rate; MSBP, mean systolic blood pressure; PT, prothrombin time; PTT, activated partial thromboplastin time; RBC, red blood cell; SD, standard deviation; WBC, white blood
